# UVB-induced nuclear translocation of TC-PTP by AKT/14-3-3σ axis inhibits keratinocyte survival and proliferation

**DOI:** 10.18632/oncotarget.21794

**Published:** 2017-10-11

**Authors:** Mihwa Kim, Liza D. Morales, Minwoo Baek, Thomas J. Slaga, John DiGiovanni, Dae Joon Kim

**Affiliations:** ^1^ Department of Biomedical Sciences, School of Medicine, University of Texas Rio Grande Valley, Edinburg, TX, USA; ^2^ South Texas Diabetes and Obesity Institute, School of Medicine, University of Texas Rio Grande Valley, Edinburg, TX, USA; ^3^ Department of Pharmacology, School of Medicine, University of Texas Health Science Center at San Antonio, San Antonio, TX, USA; ^4^ Division of Pharmacology & Toxicology, College of Pharmacy, The University of Texas at Austin, Austin, TX, USA

**Keywords:** TC-PTP, nuclear translocation, AKT, 14-3-3σ, keratinocytes

## Abstract

Understanding protein subcellular localization is important to determining the functional role of specific proteins. T-cell protein tyrosine phosphatase (TC-PTP) contains bipartite nuclear localization signals (NLSI and NLSII) in its C-terminus. We previously have demonstrated that the nuclear form of TC-PTP (TC45) is mainly localized to the cytoplasm in keratinocytes and it is translocated to the nucleus following UVB irradiation. Here, we report that TC45 is translocated by an AKT/14-3-3σ-mediated mechanism in response to UVB exposure, resulting in increased apoptosis and decreased keratinocyte proliferation. We demonstrate that UVB irradiation increased phosphorylation of AKT and induced nuclear translocation of 14-3-3σ and TC45. However, inhibition of AKT blocked nuclear translocation of TC45 and 14-3-3σ. Site-directed mutagenesis of 14-3-3σ binding sites within TC45 showed that a substitution at Threonine 179 (TC45/T179A) effectively blocked UVB-induced nuclear translocation of ectopic TC45 due to the disruption of the direct binding between TC45 and 14-3-3σ. Overexpression of TC45/T179A in keratinocytes resulted in a decrease of UVB-induced apoptosis which corresponded to an increase in nuclear phosphorylated STAT3, and cell proliferation was higher in TC45/T179A-overexpressing keratinocytes compared to control keratinocytes following UVB irradiation. Furthermore, deletion of TC45 NLSII blocked its UVB-induced nuclear translocation, indicating that both T179 and NLSII are required. Taken together, our findings suggest that AKT and 14-3-3σ cooperatively regulate TC45 nuclear translocation in a critical step of an early protective mechanism against UVB exposure that signals the deactivation of STAT3 in order to promote keratinocyte cell death and inhibit keratinocyte proliferation.

## INTRODUCTION

T-cell protein tyrosine phosphatase, or TC-PTP, (encoded by *PTPN2*) is one of 17 intracellular, non-receptor PTPs that is ubiquitously expressed in embryonic and adult tissues, however high abundance is observed in hematopoietic tissues [1, 2]. TC-PTP has been shown to have a variety of functions. Substrates targeted by TC-PTP include Janus kinase 1 (JAK1), JAK3, signal transducer and activator of transcription 1 (STAT1), STAT3, and STAT5, which suggests that TC-PTP negatively regulates the JAK/STAT signaling, an important mechanism for the transmission of extracellular signals that can regulate proliferation, differentiation, and apoptosis [3, 4]. Studies performed using TC-PTP knockout mice showed its critical role in hematopoiesis and immune function because TC-PTP knockout mice were severely defective in the hematopoietic compartment and all homozygous mice died between 3 and 5 weeks of age due to diarrhea, splenomegaly, lymphadenopathy, and anemia [5]. TC-PTP is also involved in the regulation of the cell cycle [6-8]. Furthermore, TC-PTP has a role in the regulation of diabetes and obesity by modulating insulin and leptin signaling [9-12]. In terms of cancer, recent studies revealed a focal deletion of *PTPN2* in human T-cell acute lymphoblastic leukemia, implying the potential role of TC-PTP as a tumor suppressor [13]. Studies also have shown that TC-PTP can suppress tumor growth by down-regulating STAT3 signaling in several types of cancers including breast cancer and colon cancer [14, 15].

Our previous studies using epidermal specific TC-PTP knockout mice indicated that TC-PTP can play a role in attenuating chemically-induced skin cancer formation by negatively regulating STAT3 and AKT signaling [16]. TC-PTP deficiency in mouse epidermis led to a significant decrease in apoptosis induced by the carcinogen 7,12-dimethylbenz [a]anthracene (DMBA) and a significant increase in epidermal thickness and hyperproliferation following treatment with the tumor promotor 12-*O*-tetradecanoylphorbol-13-acetate (TPA). TC-PTP knockout mice showed a shortened latency of tumorigenesis and significantly increased tumor development during DMBA/TPA skin carcinogenesis compared to control mice. Papillomas formed on TC-PTP knockout mice during carcinogenesis exhibited higher levels of phosphorylated STAT3 and phosphorylated AKT when compared with control [16].

In addition, TC-PTP is involved in the rapid dephosphorylation of STAT3 in keratinocytes which is induced by UVB irradiation. Knockdown of TC-PTP in keratinocytes resulted in a significantly higher level of phosphorylated STAT3 in the absence or presence of UVB compared with control keratinocytes [17]. Further studies revealed that TC-PTP is activated in response to UVB irradiation, which can lead to suppression of keratinocyte survival and proliferation through the down-regulation of STAT3 signaling. TC-PTP deficiency in keratinocytes increased cell proliferation and decreased apoptosis after exposure to UVB which corresponded with increased levels of phosphorylated STAT3. Similarly, overexpression of TC-PTP in keratinocytes led to a significant decrease in cell proliferation with a decrease in the level of phosphorylated STAT3 following UVB irradiation [18], suggesting a critical role for TC-PTP in the STAT3-dependent regulation of keratinocyte survival and proliferation.

There are two forms of TC-PTP generated by alternative splicing at the 3’ end of the gene: TC45 (TC-PTPa) and TC48 (TC-PTPb). The different C-termini of the isoforms determine substrate specificity and subcellular localization. TC45 (45 kDa) is the major form of TC-PTP in most species including human and mouse. TC45 contains a bipartite nuclear localization signal (NLS) and is found primarily in the nucleus, while TC48 (48 kDa) contains a hydrophobic C-terminus and is localized to the endoplasmic reticulum. [19-21]. However, our previous studies showed that TC45 is primarily localized in the cytoplasm of keratinocytes and then is translocated from the cytoplasm to the nucleus in response to UVB irradiation [17]. In the current study, we report a novel mechanism by which TC45 nuclear translocation occurs in keratinocytes *via* AKT/14-3-3σ-mediated signaling and we describe its effects on keratinocyte survival and proliferation upon exposure to UVB.

## RESULTS

### UVB-induced AKT activation triggers nuclear translocation of TC45 in keratinocytes

**Figure 1:**
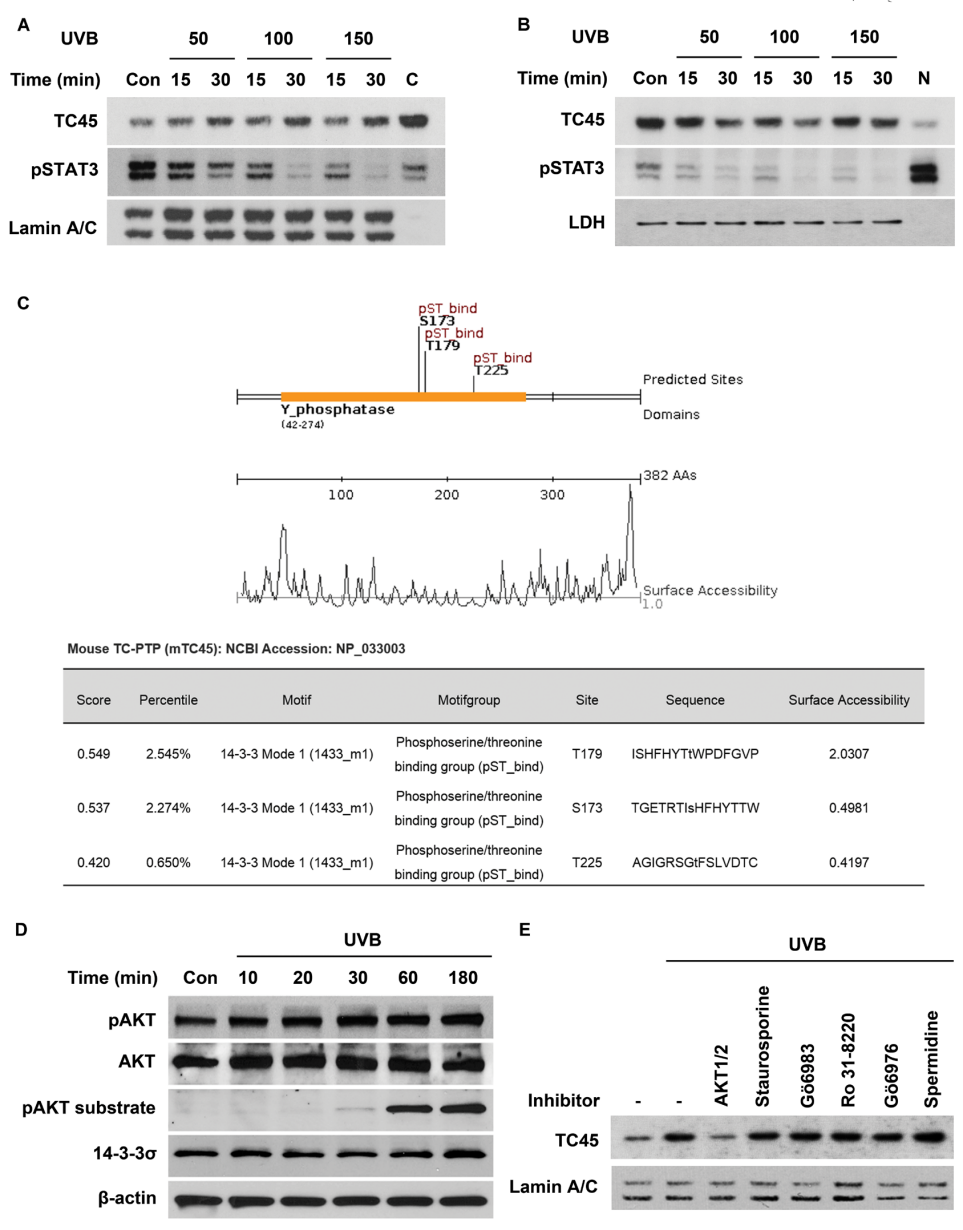
AKT regulates UVB-mediated nuclear translocation of TC45 in keratinocytes **A.** and **B.** Mouse primary keratinocytes were cultured and irradiated with UVB at three doses: 50, 100, or 150 mJ/cm^2^. Cells were collected at the indicated times after UVB exposure. Nuclear and cytoplasmic lysates were separately extracted. **A.** Western blot analysis of nuclear TC45 and phosphorylated STAT3 (pSTAT3) after UVB irradiation in keratinocytes. C: cytoplasmic fraction, no UVB irradiation. Lamin A/C was used as a control for nuclear fraction. **B.** Western blot analysis of cytoplasmic TC45 and pSTAT3 after UVB irradiation in keratinocytes. N: nuclear fraction, no UVB irradiation. LDH was used as a control for cytoplasmic fraction. **C.** Schematic representation of putative interaction motifs for 14-3-3 within the TC45 amino acid sequence identified by Scansite3 (http://scansite3.mit.edu). **D.** Western blot analysis of phosphorylated AKT (pAKT), AKT, phosphorylated AKT substrate, or 14-3-3σ in response to UVB irradiation. 3PC keratinocytes were cultured and irradiated with UVB at a dose of 100 mJ/cm^2^. Cells were collected at the indicated times. **E.** Western blot analysis of nuclear TC45 in response to UVB irradiation. Cells were cultured and treated with AKT1/2 inhibitor (1 μM), Staurosporine (10 nM), Gö6983 (1 μM), RO-31-8220 (3 μM), or Gö6976 (1 μM) for 1 h before UVB irradiation (100 mJ/cm^2^, lanes 2-8). For activation of TC45 (lane 8), cells were treated with spermidine (0.2 μM), a known TC-PTP activator, for 1 h before UVB irradiation. Cells were then collected 3 h after UVB irradiation and nuclear lysates were isolated.

Although TC45 has previously been reported to be mainly localized in the cell nucleus due to its bipartite nuclear localization signal (NLS), our recent studies indicated that TC45 is predominantly localized in the cytoplasm of skin keratinocytes and it is translocated to the nucleus following UVB exposure, suggesting there is a skin specific mechanism of TC45 nuclear translocation [17]. To identify the mechanism whereby UVB irradiation triggers nuclear translocation of TC45 in keratinocytes, mouse primary keratinocytes were exposed to three different doses of UVB (50, 100, or 150 mJ/cm^2^) and the levels of TC45 and phosphorylated STAT3 were determined in both nuclear and cytoplasmic lysates. Following UVB irradiation, TC45 was rapidly translocated to the nucleus in a dose- and time-dependent manner (Figure 1A). The level of phosphorylated STAT3 was decreased in correlation with the increase in TC45 nuclear translocation. Similarly, the level of cytoplasmic TC45 was decreased with its nuclear translocation following UVB irradiation in a dose- and time-dependent manner (Figure 1B). The level of phosphorylated STAT3 was rapidly reduced in the cytoplasm of keratinocytes following UVB exposure as previously observed [17]. To verify that UVB radiation stimulates transport of TC45 to the nucleus rather than up-regulating TC45 expression, primary keratinocytes were pretreated with cycloheximide, a protein synthesis inhibitor, before UVB irradiation. Cycloheximide had no effect on TC45 expression even in the presence of UVB irradiation (Supplementary Figure 1).

It is possible that TC45 may interact with other cytoplasmic proteins in order for it to undergo nuclear translocation. Based on this hypothesis, we searched protein-protein interaction motifs within TC45 using Scansite (http://scansite3.mit.edu) to find a protein(s) that could be involved in the process of TC45 nuclear translocation. This search revealed that TC45 contains three putative 14-3-3 binding sites (S173, T179, and T225) (Figure 1C). The 14-3-3 proteins are a family of evolutionarily conserved regulatory chaperone molecules involved in many physiological functions including signal transduction, stress response, apoptosis, and cell cycle checkpoint regulation and they function mainly by interacting with phosphoproteins containing the consensus motif RX(Y/F)XpS/TXP or RSXpS/TXP, where X represents any amino acid and pS/T represents phosphoserine and phosphothreonine, respectively [22-24]. In mammals, there are seven isoforms of 14-3-3 protein: β, ε, γ, ζ, η, σ, and τ. Most of the 14-3-3 isoforms are widely expressed in various tissues, however, 14-3-3σ is only expressed in epithelial cells [25]. 14-3-3 plays a role in a variety of cellular responses as a regulator of the subcellular localization of interacting proteins [25, 26]. AKT is one kinase capable of phosphorylating the 14-3-3 binding motifs of various target proteins including p27^Kip1^, FoxO3, and Notch5, and regulating their nucleocytoplasmic transport [27-29]. Therefore, we examined the levels of phosphorylated (active) AKT and 14-3-3σ following UVB irradiation of the 3PC mouse keratinocyte cell line, an immortalized cell line derived from DMBA-altered adult mouse primary keratinocytes which has been extensively utilized in our previous studies [16-18]. The level of AKT phosphorylation in keratinocytes gradually increased over time in response to UVB irradiation, which correlated with a subsequent increase in phosphorylated AKT substrate 30 minutes after exposure (Figure 1D, see also Supplementary Figure 2). 14-3-3σ was constitutively expressed in keratinocytes independent of UVB irradiation. To investigate whether AKT is involved in UVB-induced nuclear translocation of TC45, keratinocytes were pretreated with different inhibitors that either inhibit AKT activation (AKT1/2) or inhibit Protein Kinase C (Staurosporine, Gӧ6983, RO-31-8220 or Gӧ6976), a negative regulator of AKT signaling [30, 31]. As shown in Figure 1E, only AKT1/2 inhibitor blocked TC45 nuclear translocation after UVB irradiation as evidenced by the decrease in nuclear TC45 expression to a level similar to untreated control. In addition, spermidine, a known activator of TC-PTP, appeared to further increase the level of nuclear TC45 after UVB irradiation in comparison to UVB treatment alone (Figure 1E). These results suggest that AKT activation is important to the nuclear translocation of TC45 following UVB irradiation which is critical to TC45 regulation.

### AKT/14-3-3σ axis is required for UVB-induced TC45 nuclear translocation in keratinocytes

**Figure 2:**
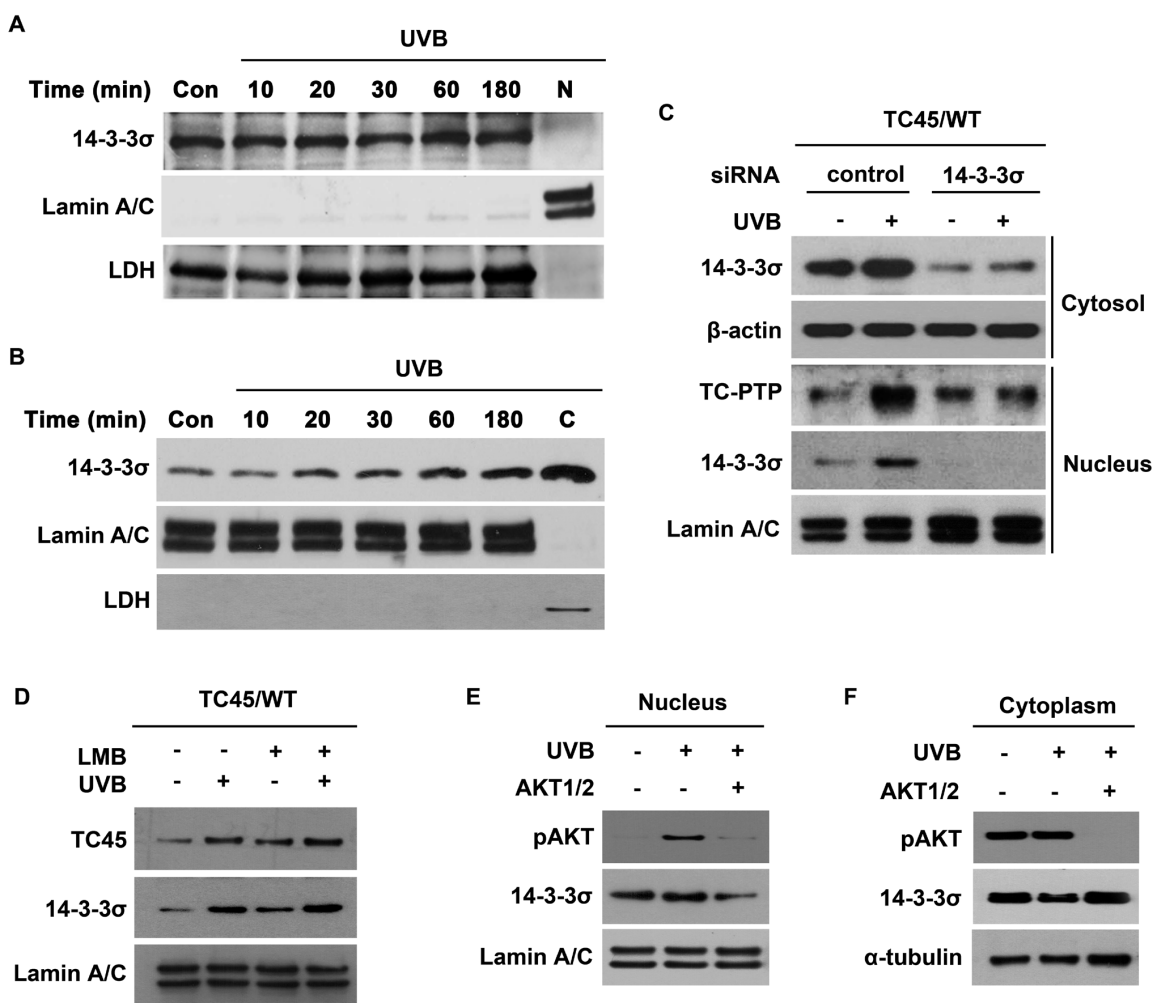
AKT cooperates with 14-3-3σ to facilitate nuclear translocation of TC45 in response to UVB irradiation Western blot analysis of **A.** cytoplasmic and **B.** nuclear 14-3-3σ in response to UVB irradiation. 3PC keratinocytes were cultured and irradiated with UVB at a dose of 100 mJ/cm^2^. Cells were collected at the indicated times after UVB exposure. Nuclear and cytoplasmic lysates were separately extracted. C: cytoplasmic fraction, no UVB irradiation. N: nuclear fraction, no UVB irradiation. **C.** 3PC keratinocytes overexpressing TC45/WT were cultured and transfected with control or 14-3-3σ-specific siRNA. Cells were collected 3 h after UVB irradiation (100 mJ/cm^2^). Nuclear and cytoplasmic fractions were isolated and resolved by SDS-PAGE and immunoblotted with antibodies specific for V5 to detect exogenous TC45, 14-3-3σ, β-actin, or Lamin A/C. **D.** 3PC keratinocytes overexpressing TC45/WT were treated with Leptomycin B (LMB) or DMSO for 1 h before UVB irradiation (100 mJ/cm^2^). Cells were collected 3 h after UVB irradiation. Nuclear fractions were isolated and resolved by SDS-PAGE and immunoblotted with antibodies specific for V5 or 14-3-3σ. Western blot analysis of **E.** nuclear pAKT and 14-3-3σ and **F**. cytoplasmic pAKT and 14-3-3σ. 3PC keratinocytes overexpressing TC45/WT were incubated with AKT1/2 inhibitor for 1 h before UVB exposure (100 mJ/cm^2^) and then cells were incubated with inhibitor again for 3 h following UVB irradiation at which time nuclear and cytoplasmic fractions were extracted.

As shown in Figure 1D, the level of 14-3-3σ expression in keratinocytes was unaffected by UVB irradiation. 14-3-3σ is an important regulatory protein with a significant number of interacting proteins that has been reported to be primarily localized in the cytoplasm of eukaryotic cells, such as U-2 OS osteosarcoma cells [32]. Therefore, we wished to confirm the subcellular localization of 14-3-3σ in keratinocytes. 14-3-3σ was primarily localized to the cytoplasm of keratinocytes (Figure 2A). Following UVB irradiation, a proportion of 14-3-3σ underwent nuclear translocation in a dose-dependent manner (Figure 2B), which is similar to the results for TC45 (Figure 1A). To investigate whether 14-3-3σ is involved in the nuclear translocation of TC45, RNAi was used to knockdown 14-3-3σ in our previously established TC45/WT-overexpressing keratinocyte cell line [18]. Knockdown of 14-3-3σ prevented TC45 nuclear translocation following UVB irradiation (Figure 2C). 14-3-3σ is known to shuttle between the cytoplasm and the nucleus [32]. In agreement with this observation, pretreatment with Leptomycin B (LMB), an inhibitor of nuclear export, further increased the level of nuclear 14-3-3σ in keratinocytes following UVB irradiation compared to untreated keratinocytes (Figure 2D). Similarly, pretreatment of keratinocytes with LMB also further increased the level of nuclear TC45 in response to UVB irradiation compared to untreated controls (Figure 2D), indicating TC45 can shuttle between the cytoplasm and the nucleus like 14-3-3σ. We also examined whether AKT phosphorylation is involved in 14-3-3σ nuclear translocation after UVB irradiation. As shown in Figure 2E and 2F, pretreatment of keratinocytes with AKT1/2 inhibitor blocked 14-3-3σ nuclear translocation in response to UVB irradiation. Collectively, these results suggest that TC45 nuclear translocation in keratinocytes is mediated by an AKT/14-3-3σ axis in response to UVB irradiation.

### Mutation at T179 within TC45 prevents its nuclear translocation in keratinocytes following UVB irradiation

**Figure 3:**
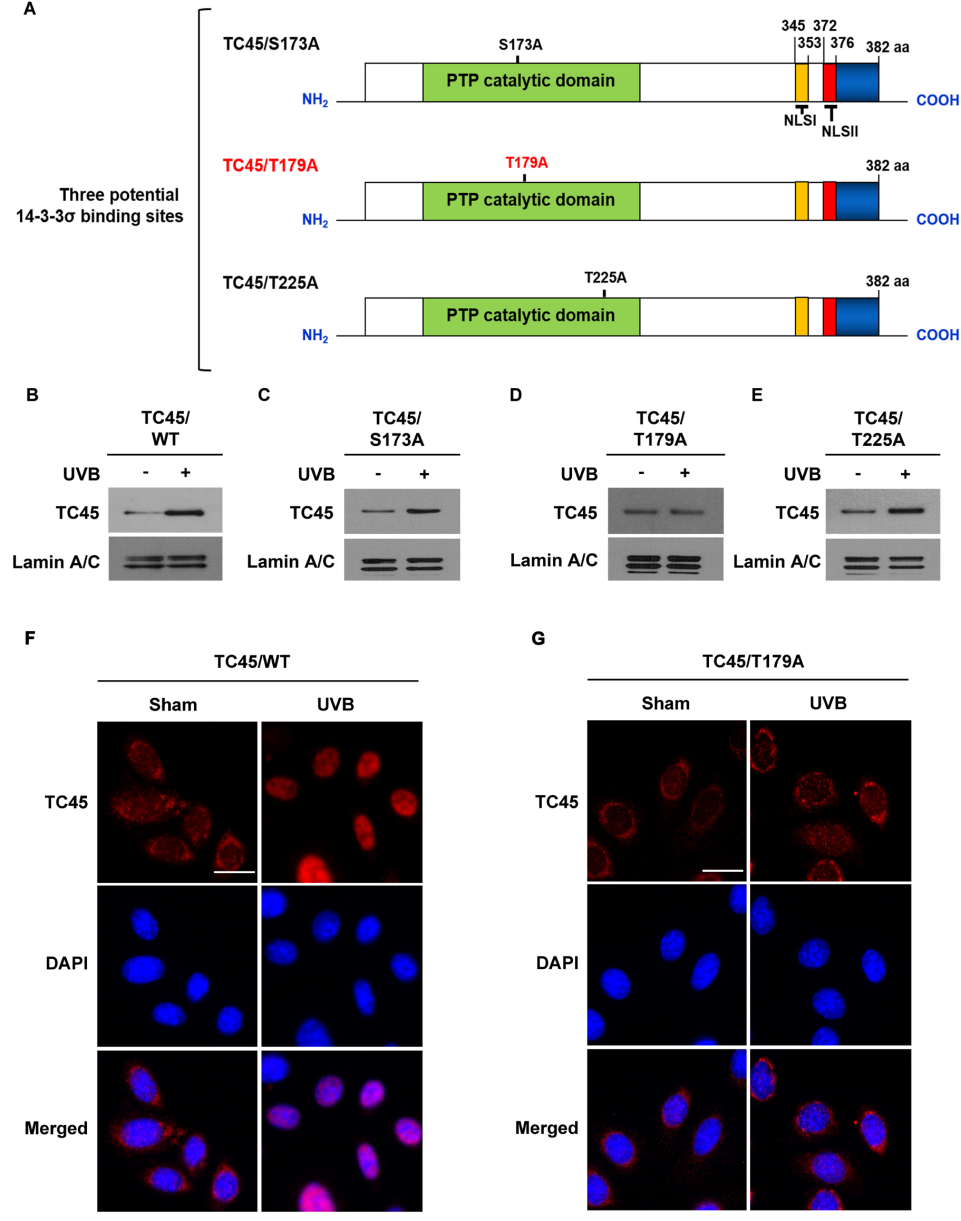
Amino acid residue Threonine 179 is required for TC45 nuclear translocation **A.** Schematic representation of site-directed mutagenesis of three putative 14-3-3σ binding sites located in the PTP catalytic domain of TC45. Serine residue at position 173 and threonine residues at positions 179 and 225 were substituted with alanine: S173A, T179A, and T225A, respectively. **B.**- **E.** Western blot analysis of nuclear TC45 in response to UVB irradiation. 3PC keratinocyte cells overexpressing **B.** TC45/WT, **C.** TC45/S173A, **D.** TC45/T179A, or **E.** TC45/T225A were cultured and irradiated with UVB at a dose of 100 mJ/cm^2^. Nuclear fractions were isolated 3 h after UVB irradiation. Exogenous TC45 expression was detected using an anti-V5 antibody. **F.** and **G.** Representative images of immunofluorescence analysis of nuclear translocation of TC45 following UVB irradiation. Keratinocytes overexpressing either **F.** TC45/WT or **G.** TC45/T179A were cultured and irradiated with UVB at a dose of 100 mJ/cm^2^. Cells were cultured for 3 h after UVB exposure and then subjected to immunofluorescence analysis by using an anti-V5 antibody to detect exogenous TC45 expression. Cells untreated with UVB were utilized as control (Sham). Scale bar: 20 μm.

As previously mentioned, TC45 contains three putative sites within its catalytic domain that are recognized as 14-3-3σ binding sites: S173, T179, and T225. To examine whether or not these sites are important for TC45 nuclear translocation, each amino acid was mutated to alanine (A) by site-directed mutagenesis (Figure 3A). Then, stable 3PC keratinocyte cell lines that overexpress each of the three mutants (S173A, T179A, or T225A) with a V5 epitope tag were generated by lentiviral transduction. Following UVB exposure, exogenous wild-type TC45 (TC45/WT) and exogenous mutants TC45/S173A and TC45/T225A underwent nuclear translocation as detected by an anti-V5-tag antibody (Figure 3B, 3C, and 3E, respectively). However, TC45/T179A was not translocated to the nucleus in response to UVB irradiation (Figure 3D). Immunofluorescent analysis clearly showed that while exogenous TC45/WT was mainly localized in the cytoplasm and then translocated to the nucleus in response to UVB irradiation (Figure 3F), exogenous TC45/T179A was primarily localized in the cytoplasm even after UVB irradiation (Figure 3G). As previously discussed, our data showed that TC45 can be prevented from shuttling between the cytoplasm and the nucleus by treatment with LMB (Figure 2D). In contrast to this result, the level of nuclear TC45/T179A was not increased in keratinocytes pretreated with LMB and irradiated with UVB (Supplementary Figure 3).

Amino acid sequence alignment of mouse TC45 with human TC45 indicates that mouse TC45 has 90% sequence identity with human TC45 and the three putative 14-3-3σ binding sites are conserved in the TC45 catalytic domain of both species (Supplementary Figure 4). To examine the nuclear translocation of human TC45 (hTC45) in keratinocytes following UVB irradiation, stable human HaCaT keratinocyte cell lines that overexpress either exogenous hTC45/WT or hTC45/T179A were generated by lentiviral transduction. Similar with mouse wild-type TC45, exogenous hTC45/WT was translocated from the cytoplasm to the nucleus after UVB irradiation. However, exogenous hTC45/T179A was not translocated to the nucleus following UVB irradiation (Supplementary Figure 5A). To confirm the involvement of 14-3-3σ in the nuclear translocation of hTC45 following UVB irradiation, RNAi was used to knockdown expression of 14-3-3σ in hTC45/WT-overexpressing HaCaT keratinocytes. Again, similar to results with mouse TC45, knockdown of 14-3-3σ resulted in the loss of exogenous hTC45 nuclear translocation following UVB irradiation (Supplementary Figure 5B). Combined, these results provide strong evidence that T179 is required for the UVB-induced nuclear translocation of TC45.

### TC45 directly interacts with 14-3-3σ in keratinocytes in response to UVB irradiation

**Figure 4:**
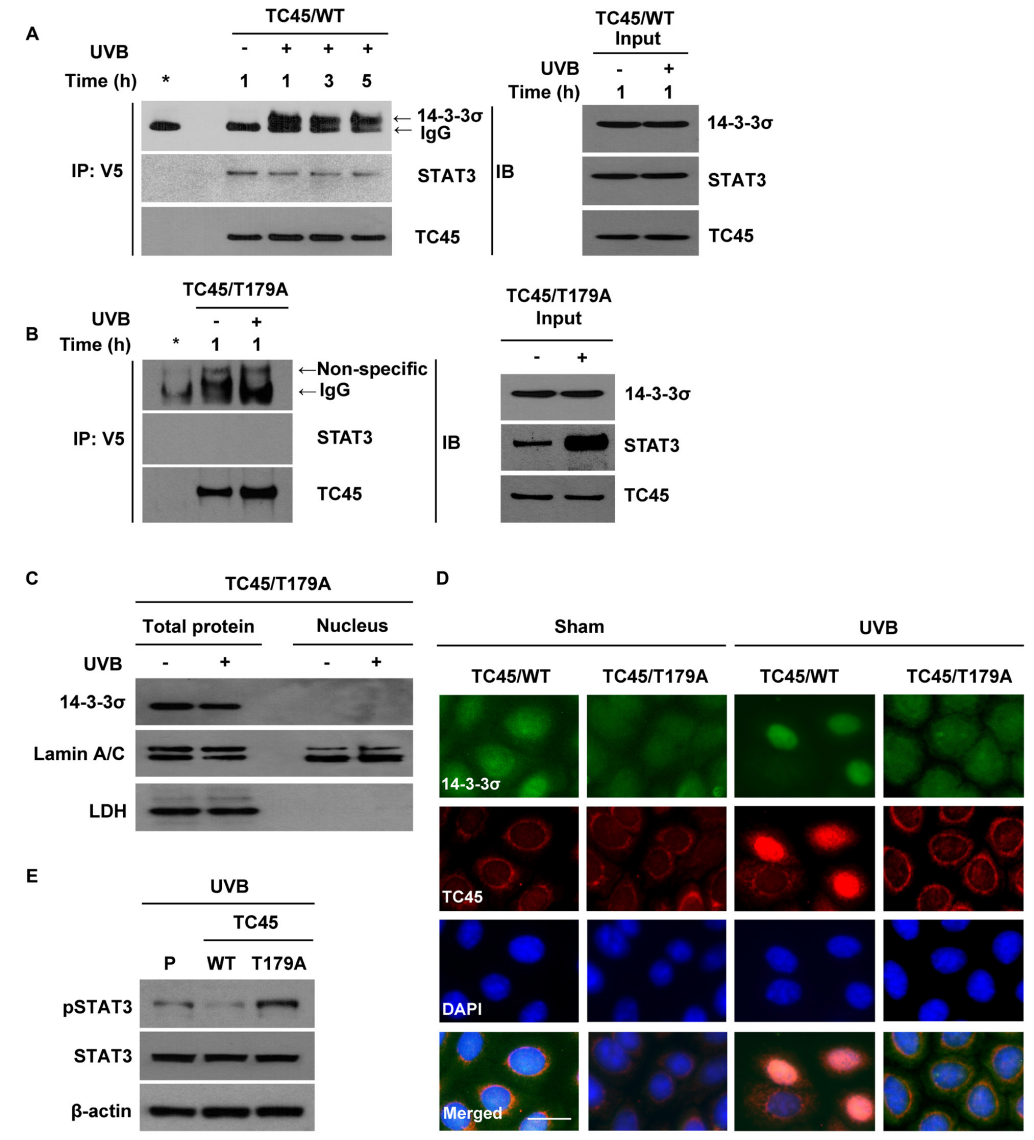
14-3-3σ transports TC45 into the keratinocyte nucleus following UVB irradiation through direct binding 3PC keratinocytes overexpressing **A.** TC45/WT or **B.** TC45/T179A were cultured and irradiated with UVB at a dose of 100 mJ/cm^2^. Cells were collected at the indicated times after UVB irradiation. Total cell lysates were subjected to immunoprecipitation with anti-V5 antibody and resolved by SDS-PAGE and immunoblotted with antibodies specific for 14-3-3σ, STAT3, or V5 (TC45). **C.** Western blot analysis of 14-3-3σ in response to UVB irradiation. 3PC keratinocytes overexpressing TC45/T179A were cultured and irradiated with UVB at a dose of 100 mJ/cm^2^. Cells were collected 3 h after UVB irradiation. Nuclear and cytoplasmic lysates were separately extracted. **D.** Representative images of immunofluorescence analysis of 14-3-3σ and TC45 following UVB irradiation. Keratinocytes overexpressing TC45/T179A were cultured and irradiated with UVB at a dose of 100 mJ/cm^2^. After 3 h of UVB irradiation, keratinocytes were subjected to immunofluorescence analysis by using an anti-V5 antibody to detect exogenous TC45 expression and an anti-14-3-3σ antibody. Scale bar: 20 μm. **E.** Western blot analysis of pSTAT3 in response to UVB irradiation. 3PC keratinocytes overexpressing TC45/WT or TC45/T179A were cultured and irradiated with UVB at a dose of 100 mJ/cm^2^. Cells were collected 3 h after UVB irradiation. Total cell lysates were then extracted.

Our results suggest that in order for TC45 to undergo nuclear translocation following UVB irradiation, AKT phosphorylates TC45, allowing TC45 to interact with 14-3-3σ through this active site. To confirm the UVB-mediated interaction of TC45 with 14-3-3σ in keratinocytes, exogenous TC45 was immunoprecipitated with an anti-V5 antibody from TC45/WT- or TC45/T179A-overexpressing 3PC cells and probed with either an anti-14-3-3σ or anti-STAT3 antibody. As shown in Figure 4A and 4B, TC45/WT interacted with 14-3-3σ following UVB irradiation, whereas TC45/T179A could not. Furthermore, TC45/WT interacted with its target STAT3 in the absence or presence of UVB irradiation. Interestingly, this interaction was not detected in TC45/T179A-overexpressing cells (Figure 4A, 4B). Then, we examined the subcellular localization of 14-3-3σ in TC45/T179A-overexpressing cells after UVB irradiation. Expression of 14-3-3σ was not detected in the nucleus of TC45/T179A-overexpressing cells in the absence or presence of UVB irradiation (Figure 4C). Immunofluorescent analysis also clearly demonstrated that while 14-3-3σ was mainly localized in cytoplasm, like TC45/WT, it is translocated to the nucleus of TC45-expressing cells in response to UVB irradiation. However, 14-3-3σ remained localized to the cytoplasm of TC45/T179A-overexpressing cells even after UVB irradiation (Figure 4D), confirming that TC45 nuclear translocation is associated with 14-3-3σ. Additionally, our data demonstrating that STAT3 interacts with TC45/WT but not with TC45/T179A implies nuclear translocation of TC45 is critical for STAT3 dephosphorylation after UVB irradiation. Correspondingly, western blot analysis showed the level of phosphorylated STAT3 was reduced in TC45/WT-expressing keratinocytes compared to control keratinocytes after UVB irradiation, whereas its expression level was increased in TC45/T179A-overexpressing keratinocytes compared to control keratinocytes in response to UVB (Figure 4E).

### Disruption of NLS II in TC45 prevents UVB-mediated nuclear translocation

**Figure 5:**
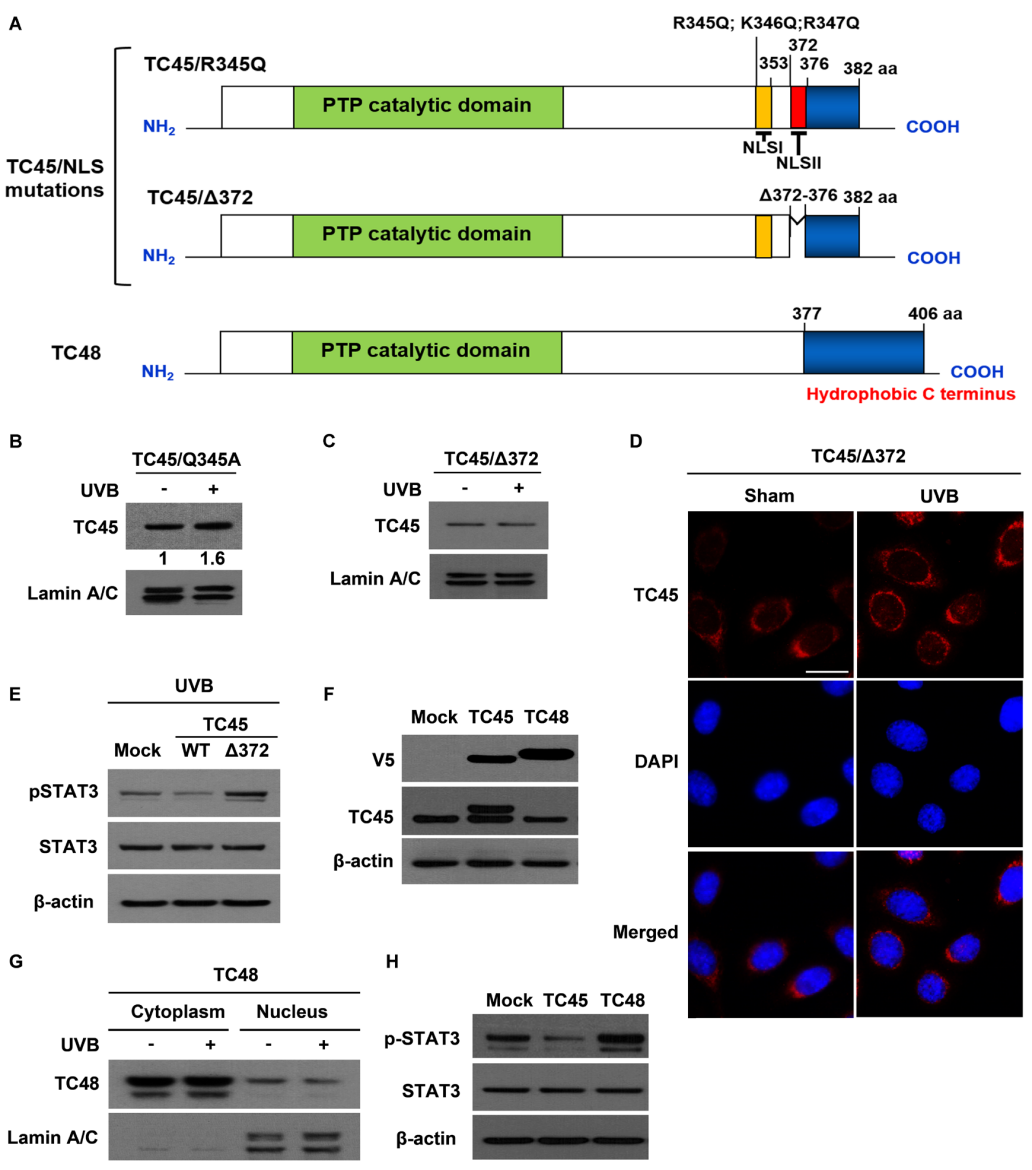
Disruption of NLSII in TC45 prevents UVB-induced nuclear translocation in keratinocytes **A.** Schematic representations of two NLS mutations, R345Q:K346Q:R347Q for NLS I and Δ372-376 for NLS II, located in the C-terminal domain of TC45 and TC48, the cytoplasmic isoform of TC-PTP. Three residues at position 345-347 were substituted with glutamine (TC45/R345Q). Five residues at positions 372-376 were deleted (TC45/Δ372). **B.** and **C.** Western blot analysis of nuclear TC45 in response to UVB irradiation. 3PC keratinocyte cells overexpressing **B**. TC45/R345Q or **C**. TC45/Δ372 were cultured and irradiated with UVB at a dose of 100 mJ/cm^2^. Nuclear fractions were isolated 3 h after UVB irradiation. Exogenous TC45 expression was detected using an anti-V5 antibody. **D.** Representative images of immunofluorescence analysis of TC45/Δ372 following UVB irradiation. Keratinocytes overexpressing TC45/Δ372 were cultured and irradiated with UVB at a dose of 100 mJ/cm^2^. After 3 h of UVB irradiation, keratinocytes were subjected to immunofluorescence analysis by using an anti-V5 antibody to detect exogenous TC45 expression. Cells untreated with UVB were utilized as control (Sham). Scale bar: 20 μm. **E.** Western blot analysis of pSTAT3 expression in response to UVB irradiation. 3PC keratinocytes overexpressing TC45/WT or TC45/Δ372 were cultured and irradiated with UVB at a dose of 100 mJ/cm^2^. Cells were collected 3 h after UVB irradiation. Total cell lysates were then extracted. **F.** Stable overexpression of TC45 or TC48 in keratinocytes. 3PC keratinocytes overexpressing TC45 or TC48 were cultured and total cell lysates were extracted. TC45 expression was detected using either an anti-V5 antibody (exogenous) or an anti-TC45 antibody (endogenous). Exogenous TC48 expression was detected using an anti-V5 antibody. **G.** 3PC keratinocytes overexpressing TC48 were cultured and irradiated with UVB at a dose of 100 mJ/cm^2^. Cells were collected 3 h after UVB irradiation and then nuclear and cytoplasmic lysates were isolated and immunoblotted with an antibody specific for V5 to detect exogenous TC48. **H.** Western blot analysis of pSTAT3 expression. 3PC keratinocytes overexpressing TC45 or TC48 were cultured Cells were collected and total cell lysates were then extracted.

Human TC45 contains a NLS in its C-terminus and consequently it is localized to the nucleus of other types of cells. Human TC45 NLS is bipartite and contains basic cluster I (residues 350-358, NLS I) and basic cluster II (residues 377-381, NLS II) [33]. It is reported that importin β, an essential factor required for nuclear transport of NLS-containing proteins, interacts with the NLSII of human TC45 and is necessary for nuclear import of TC45 [34]. In mouse, both NLS I (residues 345-353) and NLS II (residues 372-376) are conserved in its C-terminus [19] (See Supplementary Figure 3). To examine whether either TC45 NLS is involved in UVB-induced nuclear translocation in keratinocytes, we generated two NLS mutants of TC45 (Figure 5A). In the NLS I mutation (named TC45/R345Q), three basic residues (R345, K346, R347) were substituted with glutamine (Q). In the NLS II mutation (named TC45/Δ372), five residues (372-376) were deleted (Δ). We then established stable keratinocyte cell lines that overexpress either TC45/R345Q or TC45/Δ372 by lentiviral transduction. The NLS I mutation did not have an effect on UVB-induced TC45 nuclear translocation (Figure 5B). However, the NLS II mutation of TC45 blocked its nuclear translocation in response to UVB (Figure 5C). Immunofluorescent analysis clearly showed that TC45/Δ372 is exclusively localized to the cytoplasm of TC45/Δ372-overexpressing keratinocytes in the absence or presence of UVB irradiation (Figure 5D), indicating that NLS II, along with the T179-containing 14-3-3σ binding motif, is required for efficient nuclear translocation of TC45. Consistent with previous results, the level of phosphorylated STAT3 was visibly higher in TC45/Δ372-overexpressing keratinocytes compared to either control or TC45/WT-overexpressing keratinocytes after UVB irradiation (Figure 5E).

As previously mentioned, TC48 contains a hydrophobic region in its C-terminus and thereby is localized to the endoplasmic reticulum (Figure 5A). The PTP catalytic domain of TC48 is identical with that of TC45 so TC48 also contains three putative 14-3-3σ binding sites. To investigate whether TC48 is translocated to the nucleus following UVB irradiation like TC45, we cloned mouse TC48 with V5 epitope tag and generated a stable 3PC keratinocyte cell line that overexpresses it by lentiviral transduction. TC48 was detected by anti-V5 antibody, but it was not detected by TC45-specific antibody (Figure 5F). TC48 was primarily localized in the cytoplasm and UVB irradiation did not have an effect on its subcellular localization (Figure 5G). Consistent with these results, the level of phosphorylated STAT3 was very high in TC48-overexpressing keratinocytes compared to either control or TC45/WT-overexpressing keratinocytes after UVB irradiation (Figure 5H), further confirming that nuclear translocation of TC45 is required for STAT3 dephosphorylation after UVB irradiation.

### Inhibition of TC45 nuclear translocation suppressed UVB-induced apoptosis in keratinocytes

**Figure 6:**
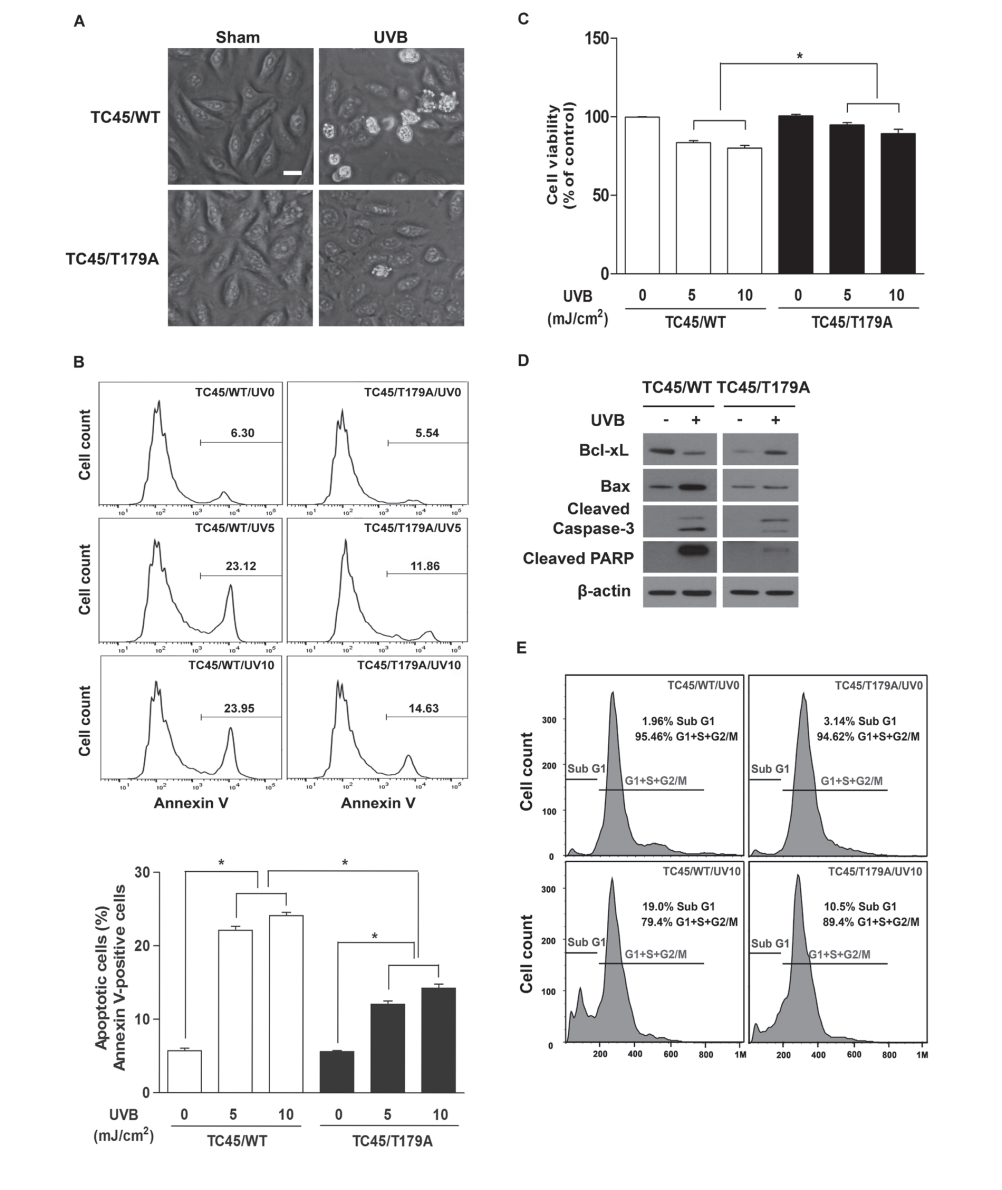
Inhibition of TC45 nuclear translocation suppresses UVB-induced apoptosis in keratinocytes **A.**. Representative images of morphological changes of keratinocytes 17 h after UVB irradiation. 3PC cells overexpressing TC45/WT or TC45/T179A were cultured and irradiated with UVB at a dose of 5 mJ/cm^2^. Scale bar: 20 μm. **B.** and **C.** Apoptosis response of TC45/WT- and TC45/T179A-overexpressing cells in response to UVB irradiation. 3PC cells overexpressing TC45/WT or TC45/T179A were irradiated with UVB at a dose of 5 or 10 mJ/cm^2^ and incubated for 17 h following UVB irradiation. Quantification of **B.** apoptotic cells stained with Annexin V and FITC and **C.** viable cells stained with PI using flow cytometry. Results are the mean ± standard deviation from three independent experiments. *p < 0.05 by *T*-test for Equality of Means. **D.** Western blot analysis of apoptosis-related proteins. 3PC cells overexpressing TC45/WT or TC45/T179A were irradiated with UVB at a dose of 5 mJ/cm^2^ and incubated for 17 h following UVB irradiation. Total cell lysates were isolated and resolved by SDS-PAGE and immunoblotted with antibodies specific for Bcl-xL, Bax, cleaved Caspase 3 (Cleaved Cas3), or cleaved PARP. **E.** Cell cycle analysis. 3PC cells overexpressing TC45/WT or TC45/T179A were irradiated with UVB at a dose of 10 mJ/cm^2^ and incubated for 24 h following UVB irradiation. DNA content was quantified through flow cytometric analysis of PI stained cells.

Studies have shown that STAT3 contributes to enhanced skin cancer formation in UVB-induced carcinogenesis by regulating its target gene expression [35]. One of the major functions of STAT3 in skin carcinogenesis is to inhibit apoptosis by upregulating anti-apoptotic gene expression. Given that inhibition of TC45 nuclear translocation results in the increase of phosphorylated STAT3 expression following UVB irradiation, we examined the impact of TC45 nuclear translocation on UVB-induced apoptosis. We utilized low dose UVB radiation in order to better observe the effect of TC45-mediated cellular responses because high dose UVB irradiation resulted in the rapid dephosphorylation of STAT3 and higher doses of UVB resulted in severe UVB-mediated damage that prevented cells from recovering [17,18]. As shown in Figure 6A, there are no morphological differences between TC45/WT-expressing cells and TC45/T179A-overexpressing cells in the absence of UVB. However, TC45/WT-expressing cells were more sensitive to low dose UVB irradiation than TC45/T179A-overexpressing cells as indicated by morphological changes. TC45/WT-expressing cells showed profound morphological changes induced by apoptosis, like rounding, bleb formation, and nuclear shrinkage, compared to TC45/T179A-overexpressing cells (Figure 6A). Consistent with this observation, flow cytometry-based apoptosis detection showed that the number of Annexin-V-positive (apoptotic) cells was significantly increased in UVB-treated TC45/WT-expressing cells compared to TC45/T179A-overexpressing cells (Figure 6B) and this result corresponded with reduced cell viability (Figure 6C). Western blot analysis showed that while the level of Bcl-xL expression, an anti-apoptotic protein regulated by STAT3, was reduced in TC45/WT-expressing cells 17 hours after low dose UVB irradiation, its expression level was increased in TC45/T179A-overexpressing cells in response to UVB (Figure 6D). Conversely, the level of Bax expression, a pro-apoptotic protein also regulated by STAT3, was clearly increased in TC45/WT-expressing cells compared to TC45/T179A-overexpressing cells after UVB irradiation. In addition, the levels of the apoptotic markers cleaved Caspase-3 and cleaved PARP were increased in TC45/WT-overexpressing cells compared to TC45/T179A-overexpressing cells in response to UVB (Figure 6D). We further examined the impact of TC45 nuclear translocation on UVB-induced apoptosis by analyzing the cell cycle using flow cytometry. Following UVB irradiation, the percentage of TC45/WT-overexpressing cells in the sub G1 phase (i.e. cells undergoing apoptosis) was much higher compared to TC45/T179A-overexpressing cells (Figure 6E). Together, these results suggest that TC45 translocation to the nucleus is a critical step in the dephosphorylation of STAT3 by TC45 which subsequently results in the promotion of apoptosis after UVB exposure.

### Inhibition of TC45 nuclear translocation promotes UVB-induced keratinocyte proliferation *via* STAT3 signaling

**Figure 7:**
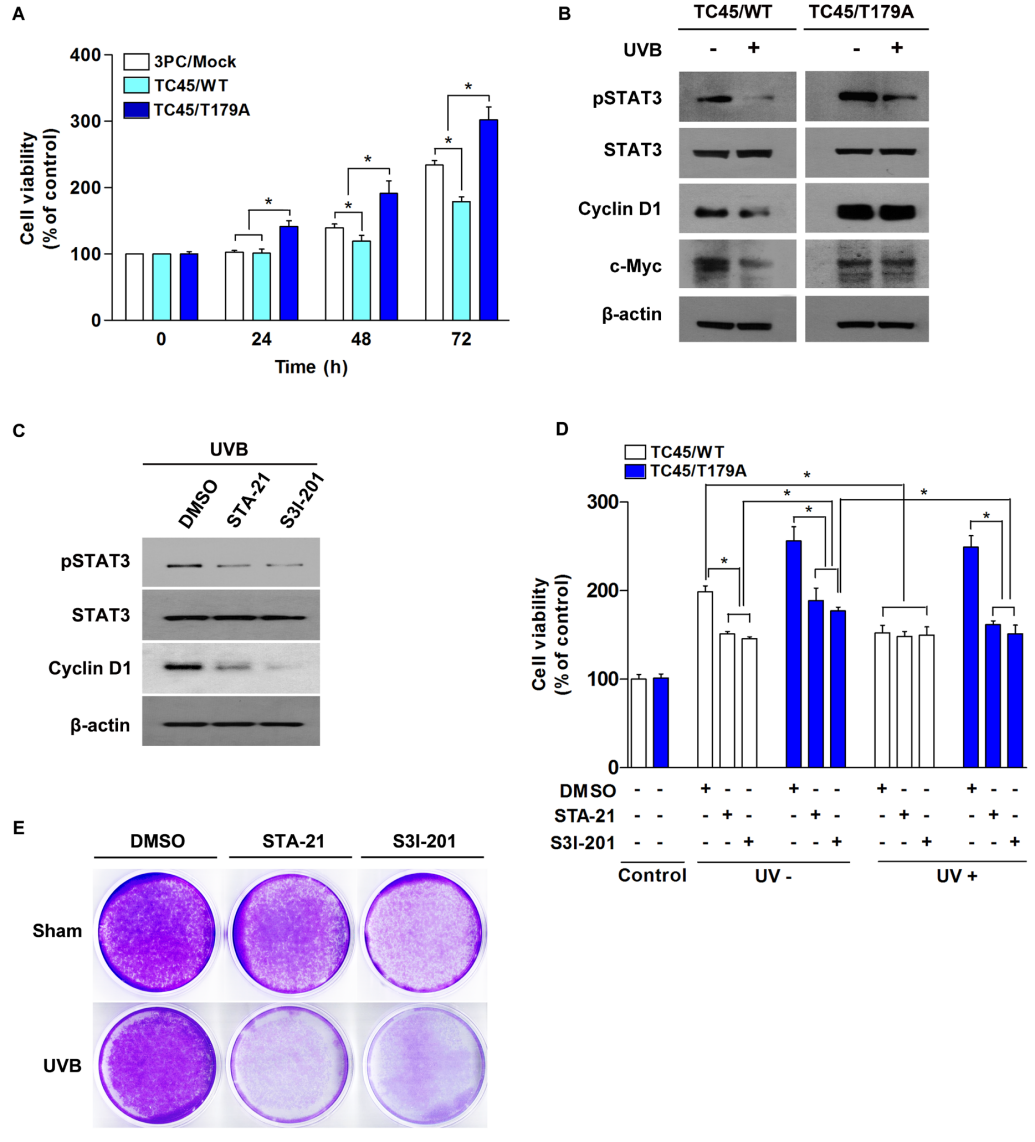
Inhibition of TC45 nuclear translocation promotes UVB-induced keratinocyte proliferation *via* STAT3 signaling **A.** Cell proliferation of TC45/WT- and TC45/T179A-overexpressing cells in response to UVB irradiation. 3PC cells overexpressing TC45/WT or TC45/T179A were cultured and irradiated with UVB at a dose of 2 mJ/cm^2^. Cell proliferation was measured by CCK-8 assay at the indicated times following UVB irradiation. Results are the mean percentage of viable cells normalized to untreated control (0 h) ± standard deviation from three independent experiments. *p < 0.05 by *T*-test for Equality of Means. **B.** 3PC cells overexpressing TC45/WT or TC45/T179A were irradiated with UVB at a dose of 5 mJ/cm^2^ and incubated for 30 min following UVB irradiation. Total cell lysates were isolated and resolved by SDS-PAGE and immunoblotted with antibodies specific for pSTAT3, STAT3, Cyclin D1, or c-Myc. **C.** Western blot analysis of pSTAT3, STAT3, Cyclin D1, or c-Myc expression in response to UVB irradiation. TC45/T179A-overexpressing cells were incubated with STAT3 inhibitors, STA21 (5µM) or S3I-201 (25 µM), for 1 h before UVB irradiation (5 mJ/cm^2^). Cells were incubated with inhibitor again for 30 min following UVB irradiation at which time total cell fractions were extracted. **D.** Effect of STAT3 inhibition on UVB-induced cell proliferation. TC45/WT- and TC45/T179A-overexpressing cells were incubated with STA21 (5 µM) or S3I-201 (25 µM) for 1 h before UVB irradiation (2 mJ/cm^2^). Cell proliferation was measured by CCK-8 assay 24 h after UVB irradiation. Results are the mean percentage of viable cells normalized to “Control” (DMSO-, UV-) ± standard deviation from three independent experiments. *p < 0.05 by *T*-test for Equality of Means. **E.** Effect of STAT3 inhibition on cell viability of TC45/T179A-overexpressing cells after UVB exposure. Cells were incubated with STA21 (5 µM) or S3I-201 (25 µM) for 1 h before UVB irradiation (5 mJ/cm^2^). Then, cells were stained with crystal violet 48 h after UVB irradiation.

In addition to the regulation of cell survival, STAT3 contributes to UVB-induced skin cancer formation by promoting cell proliferation [35]. We examined the impact of TC45 nuclear translocation on low dose UVB-induced cell proliferation. Cell viability of TC45/T179A-overexpressing 3PC cells was unaffected by low dose UVB irradiation as evidence by the fact that cell viability for TC45/T179A-overexpressing cells was significantly higher in comparison to control 3PC cells (3PC/Mock) or TC45/WT-overexpressing 3PC cells and cell viability increased over time (Figure 7A). 3PC cells overexpressing TC45/WT showed significantly reduced cell viability compared to control, indicating, again, that TC45/WT-overexpressing cells are more susceptible to low dose UVB-induced damage. Consistent with these results, Western blot analysis showed that the levels of cyclin D1 and c-Myc, STAT3 target proteins associated with cell proliferation, were reduced in TC45/WT-overexpressing cells after UVB irradiation compared to TC45/T179A-overexpressing cells (Figure 7B), implying that disruption of TC45 nuclear translocation prevents STAT3 dephosphorylation by TC45 which results in STAT3-mediated cell proliferation. To determine the impact of STAT3 on enhanced cell proliferation observed in TC45/T179A-overexpressing 3PC cells following UVB irradiation, TC45/T179A-overexpressing cells were treated with STAT3-specific inhibitors, STA-21 or S31-201, before UVB irradiation. Pretreatment with either STA-21 or S3I-201 before UVB exposure reduced the level of cyclin D1 expression in TC45/T179A-overexpressing cells (Figure 7C). We also examined keratinocyte viability in the presence of the STAT3 inhibitors. In the absence of UVB, treatment with either STA-21 or S3I-201 significantly reduced cell viability in both TC45/WT- and TC45/T179A-overexpressing cells in comparison to DMSO-treated controls (Figure 7D). Again, our results demonstrated that in the presence of UVB, cell viability of TC45/WT-overexpressing cells was significantly reduced compared to untreated control cells, and UVB irradiation had no effect on the viability of TC45/T179A-overexpressing cells as there was no significant change in cell viability of DMSO-treated control cells upon UVB exposure. However, pretreatment with either STA-21 or S3I-201 before UVB exposure significantly reduced the viability of TC45/T179A-overexpressing cells to a level comparable with that of UVB-treated TC45/WT-overexpressing cells (Figure 7D). Crystal violet staining of TC45/T179A-overexpressing cells also clearly showed that treatment with STAT3-specific inhibitors dramatically reduced cell viability 48 h after UVB irradiation compared to untreated controls (Figure 7E).

Overall, our results indicate that UVB-induced TC45 nuclear translocation by the AKT/14-3-3σ axis allows for the dephosphorylation of STAT3, leading to increased apoptosis and decreased cell proliferation of keratinocytes, which can contribute to the prevention of skin cancer development.

## DISCUSSION

**Figure 8:**
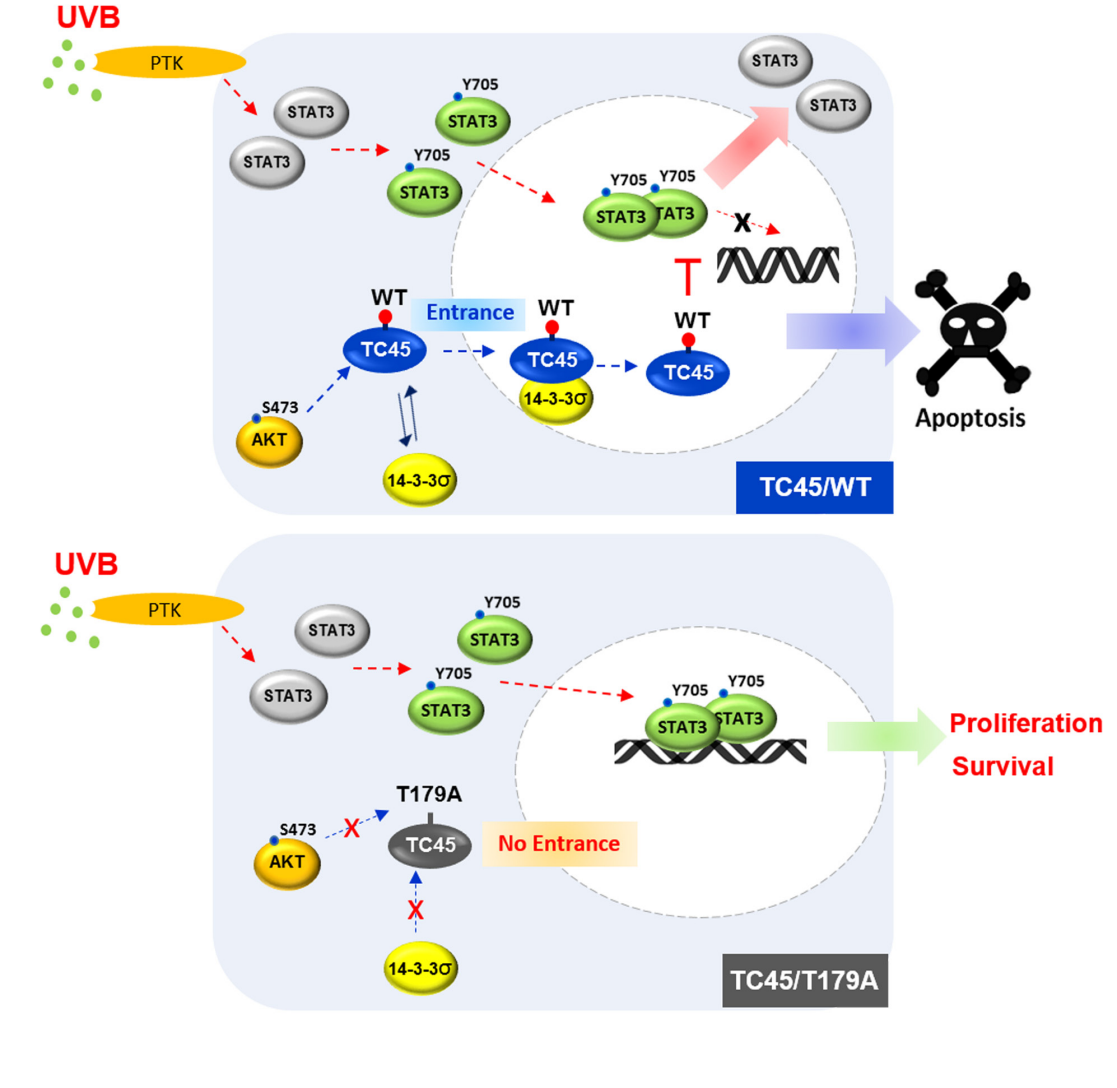
Schematic diagram of the mechanism of TC45 nuclear translocation in keratinocytes following UVB irradiation UVB irradiation triggers AKT activation which promotes nuclear translocation of TC45 with 14-3-3σ. AKT-mediated phosphorylation of T179 within the catalytic domain of TC45 and the subsequent binding of 14-3-3σ at this site facilitate the translocation of cytoplasmic TC45 to the nucleus upon UVB exposure. Once TC45 is translocated to the nucleus, it is capable of interacting with active STAT3 to dephosphorylate it, resulting in the promotion of apoptosis and the inhibition of keratinocyte cell proliferation.

PTPs play important roles in various physiological functions including growth, differentiation, apoptosis, and motility by negatively regulating the rate and duration of phosphotyrosine signaling [36, 37]. It has been recognized that subcellular localization and translocation of PTPs are critical in regulating their physiological functions by making it possible to access their target substrates [38]. Subcellular localization and translocation of PTPs are regulated by their unique domains, such as NLS, or by phosphorylation. For example, a non-receptor form of PTPε is localized to the nucleus by its unique N-terminal domain [39]. PTP36/PTPD2/Pez, one of the non-transmembrane PTPs, is localized to the cytoplasm by phosphorylation of its serine residues. Moreover, PTP36 is translocated from the cytoplasm to the cytoskeleton by dephosphorylation [40]. TC45, a major form of TC-PTP, has been reported to primarily localize to the nucleus of cells by its bipartite NLS [19]. In contrast, our recent studies showed keratinocyte-specific localization of TC45 in that TC45 is primarily localized in the cytoplasm of keratinocytes and then is translocated from the cytoplasm to the nucleus in response to UVB irradiation [16]. In the current study, we describe a novel mechanism for TC45 nuclear translocation induced by UVB exposure. UVB irradiation triggers AKT activation in keratinocytes. Then, AKT-mediated phosphorylation of T179 in the catalytic domain of TC45 and the subsequent direct interaction of TC45 with 14-3-3σ promotes the translocation of cytoplasmic TC45 to the nucleus in response to UVB irradiation. Translocation of TC45 allows it to interact with active/phosphorylated STAT3 in the nucleus and dephosphorylate it, resulting in the promotion of UVB-induced apoptosis and the inhibition of keratinocyte cell proliferation (Figure 8).

STAT3 is one of a family of cytoplasmic proteins that participates in normal cellular responses to cytokines and growth factors as a transcription factor. STAT3 modulates various physiological functions including apoptosis, cell cycle regulation, and tumor angiogenesis through regulation of gene expression, and its constitutive activation is associated with a number of human epithelial cancers [41-43]. Recent studies have shown that activation of STAT3 might be a predominant factor in the development of UVB-induced skin tumors by promoting keratinocyte survival and proliferation [35, 44, 45]. TC45 has been reported to play a role in cell cycle regulation and an Interleukin-6-mediated signaling pathway by targeting STAT3 [46-48]. In contrast to other types of tissues, TC45 is primarily localized in the cytoplasm of keratinocytes (Figure 1A, 1B). Our studies suggest that TC45 nuclear translocation by the AKT/14-3-3σ axis is critical for the regulation of STAT3 signaling. Inhibition of TC45 nuclear translocation *via* T179A mutation or NLSII deletion significantly increased the level of phosphorylated STAT3 in keratinocytes following UVB irradiation compared to control (Figures 4A, 5E). Consistent with these results, blocking TC45 nuclear translocation with the T179A mutation also reduced UVB-induced apoptosis (Figure 6) and promoted cell proliferation (Figure 7). These results further demonstrate that subcellular localization and translocation of PTPs are crucial for the regulation of their cellular functions.

AKT is a serine/threonine kinase that is associated with a variety of cancers given its ability to phosphorylate proteins involved in cell survival, metabolism, and cell cycle progression [49, 50]. In this regard, it has been suggested that AKT is a critical oncogenic factor in skin carcinogenesis [51]. Mice overexpressing either epidermal specific AKT or constitutively active AKT showed a significantly increased sensitivity to two-stage skin carcinogenesis [52]. Recent studies also showed that epidermal specific TC-PTP knockout mice had a significantly increased sensitivity to two-stage skin carcinogenesis which correlated with higher levels of phosphorylated STAT3 and phosphorylated AKT expression [16]. In addition, AKT activation induced by UV irradiation has been shown to prevent cell death and promote cell proliferation in the skin [53]. Our current studies indicate that AKT plays a role in TC45-mediated dephosphorylation of STAT3 through its ability to facilitate TC45 translocation in response to UVB irradiation. Inhibition of AKT prevented nuclear translocation of TC45 and 14-3-3σ following UVB exposure (Figures 1E, 2E), leading to increased STAT3 phosphorylation, which can contribute to the promotion of keratinocyte survival and proliferation. Our results suggest that AKT can play two roles in the keratinocyte response to UVB radiation: (1) AKT can participate in a protective mechanism that is triggered after initial exposure to UVB which initially prohibits keratinocyte proliferation and survival [18] to minimize potential UVB damage and (2) following prolonged or excessive exposure to UVB radiation, aberrant AKT activation can promote keratinocyte survival and proliferation, leading to skin carcinogenesis. Since TC45 can inhibit AKT signaling [16], it is possible that once TC45 has been transported to the nucleus, a negative feedback loop could arrest AKT-mediated TC45 nuclear translocation. In this case, levels of phosphorylated STAT3 would recover, which we previously have observed [18], and keratinocyte proliferation would proceed normally.

In conclusion, our findings provide a description of a novel mechanism induced by UVB irradiation that allows for TC45 nuclear translocation by the AKT/14-3-3σ axis and that plays a significant part in an initial protective mechanism against UVB exposure that allows for the efficient removal of UVB-damaged cells by increasing apoptosis and reducing cell proliferation through the dephosphorylation of STAT3.

## MATERIALS AND METHODS

### Cell culture and UVB irradiation

Primary keratinocytes obtained from 2 day-old neonates from wild-type FVB mice through a previously described method [54] and were cultured in keratinocyte growth medium containing 1% fetal bovine serum (FBS) and 1% penicillin/streptomycin at 37 °C and 5% CO_2_. The 3PC mouse cell line is an immortalized, benign keratinocyte cell line that was derived from adult mouse primary keratinocytes after exposure *in vitro* to 7,12-dimethylbenz [a]anthracene and selection with high Ca^2+^ medium [55]. 3PC cells were cultured in EMEM without CaCl_2_ (Lonza) containing 0.03 mM Ca^2+^, 1% FBS, 1% penicillin/streptomycin, 5 ng/ml EGF, 2.5 μg/ml insulin, 5 μg/ml hydrocortison, 10 μg/ml transferrin, 10 μM ethanolamine, and 10 μM phosphoethanolamine. HaCaT keratinocytes (CLS-300493, CLS Cell Lines Service GmbH, Eppelheim, Germany; immortalized and nontumorigenic human skin keratinocyte cell line) [56] were cultured in DMEM containing 10% FBS and 1% penicillin/streptomycin at 37 °C and 5% CO_2_. Keratinocyte cell lines were verified by consistent morphological characteristics and used within 5 passages for experiments. Keratinocytes were cultured to 80 - 85% confluence, at which time cells were washed with DPBS. A small volume of DPBS was added to coat keratinocytes with a thin layer of DPBS, and then keratinocytes were irradiated with UVB. Immediately after irradiation, DPBS was removed, and prewarmed medium was added to cells for additional culture time before harvest.

### Establishment of TC-PTP or mutant TC-PTP overexpressing 3PC cell lines

Stable mouse 3PC keratinocyte cell lines overexpressing TC45 mutants (referred to as TC45/S173A, TC45/T179A, TC45/T225A, TC45/Q345A, or TC45/Δ372) with a V5 epitope tag at the C-terminus were established by lentiviral transduction using the lentivirus entry vector pEF-ENTR B (Addgene plasmid #17428) and the destination vector pLenti X1 Puro DEST (Addgene plasmid #17297) [57]. The entry vector overexpressing mouse TC45 (referred to as TC45/WT) [18] was mutated using the QuikChange^®^ site-directed mutagenesis kit (Stratagene) to generate each of the TC45 mutants using corresponding site-specific mutant primers (Table 1). Gateway^®^ LR Clonase^®^ enzyme mix (Invitrogen) was used to catalyze an LR recombination reaction between the entry vectors expressing TC45 mutants and the destination vector to generate the final lentiviral expression clones for TC45 mutants, respectively. Then, 3PC keratinocytes were infected with lentiviral particles from the lentiviral expression clones and selected with puromycin. Wild-type human TC45 (referred to as hTC45/WT) cDNA from pCMV-SPORT6-hTC45 (Open Biosystems) was amplified by PCR and cloned into the lentivirus entry vector pEF-ENTR B. The entry vector containing hTC45/WT was mutated using the QuikChange^®^ site-directed mutagenesis kit to generate an hTC45 mutant clone containing the substitution T179A using site-specific mutant primers (Table 1). Stable human HaCaT keratinocyte cell lines overexpressing hTC45/WT or hTC45/T179A with V5 epitope tag were established by lentiviral transduction as described above. Wild-type mouse TC48 (referred to as TC48/WT) cDNA from pCMV-SPORT6-mTC48 (Open Biosystems) was amplified by PCR and cloned into the lentivirus entry vector pEF-ENTR B. A stable mouse 3PC keratinocyte cell line overexpressing TC48/WT with V5 epitope tag was established by lentiviral transduction as described.

**Table 1:** Oligonucleotide primers for site-directed mutagenesis of TC45

Species	Clone name	Target mutation site	Oligonucleotide pimers		Substitution
		Amino acid	Sequence (5'→3')		Single letter Amino acid code
Human	TC45/T179A	179	Forward	cca gaa caa tat ctc act ttc att ata ctg cct ggc cag att ttg	T179 → A179
			Reverse	caa aat ctg gcc agg cag tat aat gaaa gtg aga tat tgt tct gg	
Mouse	TC45/S173A	173	Forward	ctg gtg aaa cca gaa cca tag ccc act tcc att ata cca cct	S173 → A173
			Reverse	agg tgg tat aat gga agt ggg cta tgg ttc tgg ttt cac cag	
Mouse	TC45/T179A	179	Forward	ata tct cac ttc cat tat acc gcc tgg cca gat ttt gg	T179 → A179
			Reverse	cca aaa tct ggc cag gcg gta taa tgg aag tga gat at	
Mouse	TC45/T225A	225	Forward	cgg gcg ctc tgg cgc ctt ctc tct tgt	T225 → A225
			Reverse	aca aga gag aag gcg cca gag cgc ccg	
Mouse	TC45/R345Q	345, 346, 347	Forward	gga gag cag tga gag cat tct aca gca aca gat tcg aga gga tag aaa ggc tac	R345Q; K346Q; R347Q
			Reverse	gta gcc ttt cta tcc tct cga atc tgt tgc tgt aga atg ctc tca ctg ctc tcc	
Mouse	TC45/Δ372	372-376	Forward	cag agg cta aat gaa act gaa cca aga ttg aca gac acc	D372-376: deletion
			Reverse	ggt gtc tgt caa tct tgg ttc agt ttc att tag cct ctg	

### Antibodies and inhibitors

The antibodies against TC-PTP (TC45, #58935), pSTAT3 (Y705) (#9145), STAT3 (#9139), pAKT (S473) (#4060), AKT (#4685), Phospho-AKT substrate (#9614), Bcl-xL (#2764), Bax (#2772), Cleaved Caspase 3 (#9661), Cleaved PARP (#9548), Cyclin D1 (2978) and α/β tubulin (#2148) were purchased from Cell Signaling Technology. The antibodies against Lamin A/C (sc-7293), c-Myc (sc-40), β-actin (sc-47778), and LDH (sc-33781) were bought from Santa Cruz Biotechnology. The antibody against 14-3-3σ (GTX100801) was purchased from GeneTex. Anti-V5 tag antibody (R960-25) was purchased from Invitrogen and used for detection of exogenous TC45. STA-21, S3I-201, AKT1/2 inhibitor, Staurosporine, Gö6983, Ro-31-8220, Gö6976, spermidine, protease inhibitor cocktail, phosphatase inhibitor cocktail I, phosphatase inhibitor cocktail II and Leptomycin B were purchased from Sigma-Aldrich.

### Immunoblot analysis

Total cell lysates were prepared with RIPA Buffer (Pierce) containing 1% Triton X-100, protease inhibitor cocktail, and phosphatase inhibitor cocktail I and II (Sigma-Aldrich). Equal amounts of total protein were resolved using SDS-PAGE and subsequently transferred to PVDF membrane (GE Healthcare). The membrane was incubated overnight with primary antibody followed by incubation with a horseradish peroxidase-conjugated secondary antibodies. Chemiluminescent detection reagents (Pierce) were used to detect immunoreactive protein.

### Immunoprecipitation analysis

Total cell lysates were prepared with Nonidet P-40-containing immunoprecipitation buffer (40 mM Tris, pH 7.4, 120 mM NaCl, 10 mM EDTA, 0.1% (v/v) Nonidet P-40) containing protease inhibitor mixture, and phosphatase inhibitor cocktail I and II. Prior to immunoprecipitation, antibodies are incubated with magnetic DynabeadsTM Protein G (Invitrogen) and then 2 mg of protein lysates was added into V5-attached magnetic beads and incubated overnight at 4 °C. After immunoprecipitation, the beads were washed three times with washing buffer and then the immune complexes were eluted from the beads and subjected to SDS-PAGE and immunoblot analysis as previously described.

### RNA interference (RNAi)

3PC keratinocytes were grown overnight to ∼40% confluence and transfected with control siRNA or 14-3-3σ-specific siRNA (Santa Cruz Biotechnology). Transfection was performed with Lipofectamine RNAiMAX (Invitrogen) according to the manufacturer’s instructions.

### Flow cytometric analysis for apoptosis and cell cycle

Apoptosis of keratinocytes was assessed by an Annexin V-FITC apoptosis kit (Molecular probe, V13242) according of the manufacturer’s instructions. Briefly, cells (1 x 10^6^) were collected at 17 h after UVB irradiation (5 or 10 mJ/cm^2^). Cells were then washed with cold PBS and centrifuged at 400 x g for 5 min and stained using Annexin V-FITC. Then, cell suspensions were analyzed using LSRFortessa (BD Biosciences). The apoptotic rate was calculated as Annexin V-positive cells. Each experiment was performed three times, and data were presented as means ± standard deviation. For cell cycle analysis, cells were exposed to UVB (5 mJ/cm^2^). Twenty four hours after UVB irradiation, cells were collected and fixed with 70% ethanol. After washing twice with cold PBS, cells were stained with propidium iodide. All of the resulting data was produced and analyzed using Flowjo V10.2 software.

### Cell proliferation assay

Keratinocyte proliferation was assessed by the Cell Counting Kit-8 (Dojindo Laboratories) colorimetric assay according to the manufacturer’s instructions. Briefly, keratinocytes were plated at a density of 2 x 10^3^ ∼ 2 x 10^4^ cells/well in 96-well cell culture plates. Then, cells were irradiated by UVB as described above and recultured for 24 - 72 h. Then, the formazan dye was added to each well and absorbance was read at 450 nm.

### Crystal violet staining

Cells (1 x 10^5^ cells/well) were cultured in the presence of STAT3 inhibitors STA-21 or S3I-201 for 1 h before UVB irradiation (5 mJ/cm^2^). Following UVB irradiation, cells were incubated for 48 h and then stained with 0.05% crystal violet.

### Immunocytochemical analysis

Cells (5 x 10^4^ cells/well) were seeded on an IbiTreat 30 µ-Dish culture plate (ibidi, GmbH). The following day, cells were irradiated with UVB and recultured for 3 h. Cells were then fixed with 4% paraformaldehyde for 15 min. Cells were washed with cold PBS and then incubated with 0.02% Triton X-100 for 20 min. After being washed again with PBS, cells were incubated with background sniper (Biocare Medical) for 1 h. Then, cells were incubated with an anti-V5 tag antibody for detection of exogenous TC45 or anti-14-3-3σ antibody and washed with PBS. Cells were incubated with Alexa Fluor 488 or 647-conjugated secondary antibodies and washed with PBS. Slides were rinsed with PBS, mounted in VECTASHIELD Mounting Medium for fluorescence with DAPI (Vector Laboratories, H-1200) and sealed with clear nail varnish. Images were taken by fluorescence microscopy.

## SUPPLEMENTARY MATERIALS FIGURES


