# Supplementary material for: UVB-induced nuclear translocation of TC-PTP by AKT/14-3-3σ axis inhibits keratinocyte survival and proliferation
Source: Oncotarget. 2017 Oct 11;8(53):90674–92. doi: 10.18632/oncotarget.21794 (PMC5710877; doi:10.18632/oncotarget.21794)
Supplement: Supplementary file 1 [file oncotarget-08-90674-s001.pdf]

UVB-induced nuclear translocation of TC-PTP by AKT/14-3-3σ axis inhibits keratinocyte survival and proliferation

SUPPLEMENTARY MATERIALS

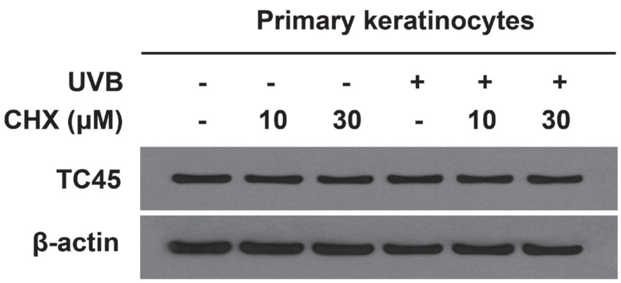

Supplementary Figure 1: UVB radiation does not induce protein synthesis of TC45. 3PC keratinocytes were cultured and treated with cyclohexamide for 2 h before UVB irradiation (100 mJ/cm^2). Cells were then collected 3 h after UVB irradiation and total cells lysates were isolated, resolved by SDS-PAGE and immunoblotted with an antibody against TC45. CHX: cyclohexamide.

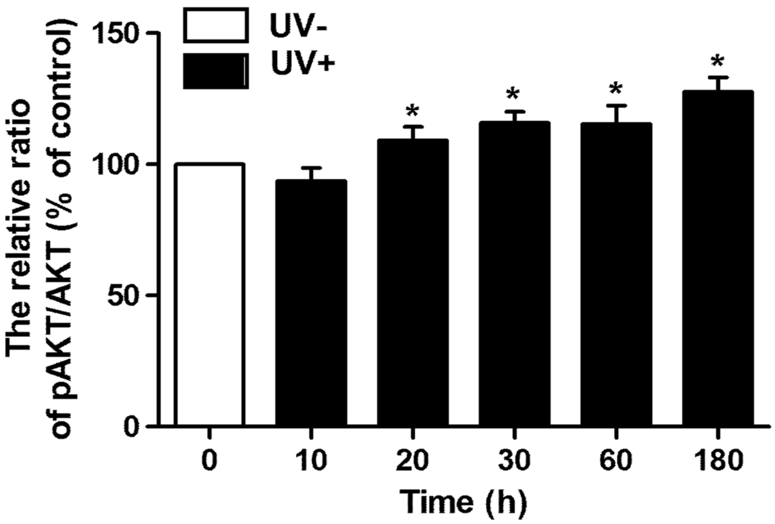

Supplementary Figure 2: Phosphorylated AKT expression following UVB irradiation. The relative ratio of phosphorylated AKT (pAKT)/AKT was quantified by densitometry. Results are the mean ± standard deviation from three independent experiments. *$p < 0.05$ by T-test for Equality of Means.

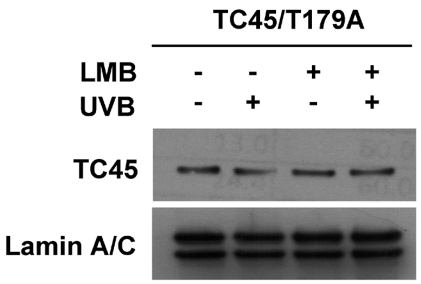

Supplementary Figure 3: T179 mutation of TC45 (TC45/T179A) blocks its nuclear translocation in keratinocytes following UVB irradiation. 3PC keratinocytes overexpressing TC45/T179A were treated with the nuclear export inhibitor Leptomycin B (LMB) or DMSO control for 1 h before UVB irradiation (100 mJ/cm[2]). Cells were collected 3 h after UVB irradiation. Nuclear fractions were isolated and resolved by SDS-PAGE and immunoblotted with antibodies specific for V5 to detect exogenous TC45.

Mouse PTPN2 (TC45): Accession NP_033003
Human PTPN2 (TC45): Accession NP_536347

Alignment with Mouse TC45 and Human TC45, Identities: 90%

```
Mouse TC45  1    MSATIEREFEELDAQCRWQPLYLEIRNESHDYPHRVAKFPENRNRNRYRDVSPYDHSRVK  60
                 M  TIEREFEELD Q RWQPLYLEIRNESHDYPHRVAKFPENRNRNRYRDVSPYDHSRVK
Human TC45  1    MPTTIEREFEELDTQRRWQPLYLEIRNESHDYPHRVAKFPENRNRNRYRDVSPYDHSRVK  60

Mouse TC45  61   LQSTENDYINASLVDIEEAQRSYILTQGPLPNTCCHFWLMVWQQKTKAVVMLNRTVEKES  120
                 LQ+ ENDYINASLVDIEEAQRSYILTQGPLPNTCCHFWLMVWQQKTKAVVMLNR VEKES
Human TC45  61   LQNAENDYINASLVDIEEAQRSYILTQGPLPNTCCHFWLMVWQQKTKAVVMLNRIVEKES  120
                                                                   173   179
Mouse TC45  121  VKCAQYWPTDDREMVFKETGFSVKLLSEDVKSYYTVHLLQLENINTGETRTISHFHYTTW  180
                 VKCAQYWPTDD+EM+FKETGFSVKLLSEDVKSYYTVHLLQLENIN+GETRTISHFHYTTW
Human TC45  121  VKCAQYWPTDDQEMLFKETGFSVKLLSEDVKSYYTVHLLQLENINSGETRTISHFHYTTW  180
                                                        225
Mouse TC45  181  PDFGVPESPASFLNFLFKVRESGCLTPDHGPAVIHCSAGIGRSGTFSLVDTCLVLMEKGE  240
                 PDFGVPESPASFLNFLFKVRESG L PDHGPAVIHCSAGIGRSGTFSLVDTCLVLMEKG+
Human TC45  181  PDFGVPESPASFLNFLFKVRESGSLNPDHGPAVIHCSAGIGRSGTFSLVDTCLVLMEKGD  240

Mouse TC45  241  DVNVKQLLLNMRKYRMGLIQTPDQLRFSYMAIIEGAKYTKGDSNIQKRWKELSKEDLSPI  300
                 D+N+KQ+LLNMRKYRMGLIQTPDQLRFSYMAIIEGAK  KGDS+IQKRWKELSKEDLSP
Human TC45  241  DINIKQVLLNMRKYRMGLIQTPDQLRFSYMAIIEGAKCIKGDSSIQKRWKELSKEDLSPA  300
                                                              345; 346; 347
Mouse TC45  301  CDHSQNRVMVEKYNGKRIGSEDEKLTG-----LPSKVQDTVEESSESILRKRIREDRKAT  355
                  DHS N++M EKYNG RIG E+EKLTG     L SK+QDT+EE+SES LRKRIREDRKAT
Human TC45  301  FDHSPNKIMTEKYNGNRIGLEEEKLTGDRCTGLSSKMQDTMEENSESALRKRIREDRKAT  360
                           372^376
Mouse TC45  356  TAQKVQQMKQRLNETERKRKRPRLTDT   382
                 TAQKVQQMKQRLNE ERKRKRPRLTDT
Human TC45  361  TAQKVQQMKQRLNENERKRKRPRLTDT   387
```

Supplementary Figure 4: Amino acid sequence alignment of mouse TC45 and human TC45. Amino acids mutated by site-directed mutagenesis are indicated in boldface. Deleted amino acids of NLSII for TC45/Δ372 are shown in the boxed area.

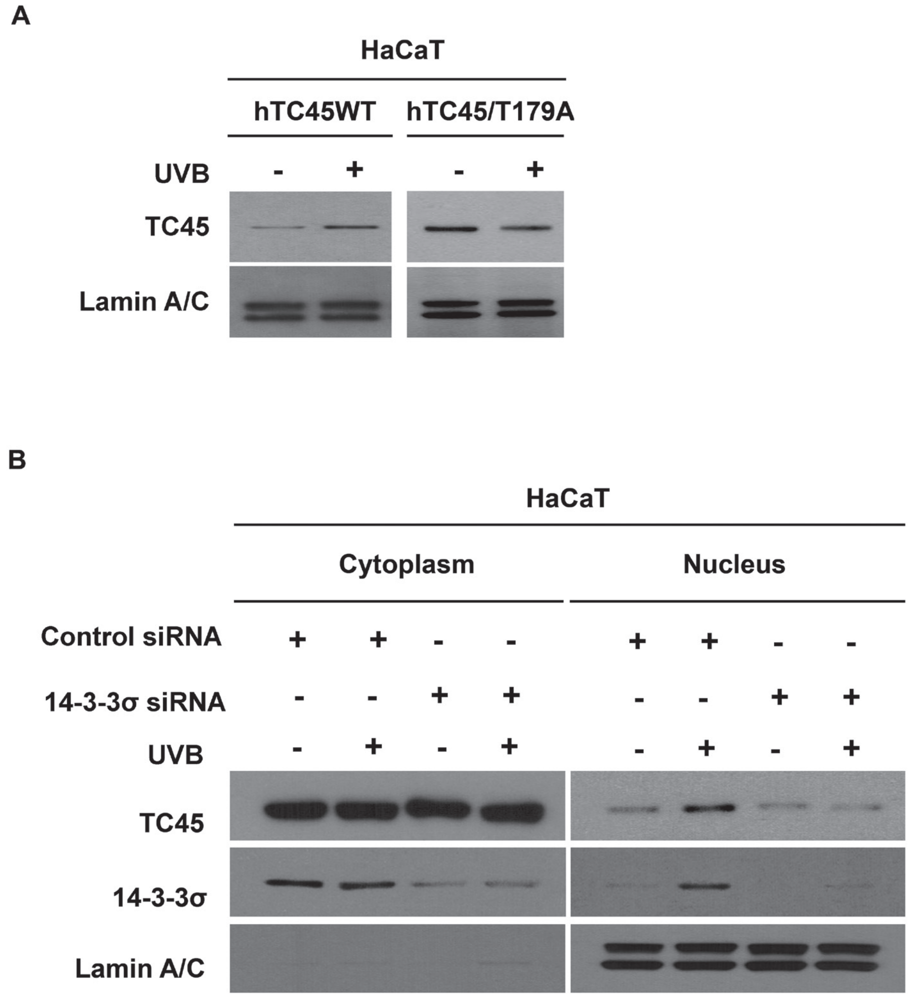

Supplementary Figure 5: 14-3-3σ mediates hTC45 nuclear translocation in keratinocytes following UVB irradiation. A. Western blot analysis of nuclear hTC45 expression following UVB irradiation. Human HaCaT keratinocyte cell lines overexpressing hTC45/WT or hTC45/T179A were cultured and collected 3 h after UVB irradiation (100 mJ/cm^2). Nuclear fractions were isolated 3 h after UVB irradiation. Exogenous TC45 expression was detected using an anti-V5 antibody. B. Knockdown of 14-3-3σ prevents UVB-induced hTC45 nuclear translocation. HaCaT keratinocytes overexpressing hTC45/WT were cultured and transfected with control or 14-3-3σ-specific siRNA. Cells were collected 3 h after UVB irradiation (100 mJ/cm^2). Nuclear and cytoplasmic fractions were isolated and resolved by SDS-PAGE and immunoblotted with antibodies specific for V5 to detect exogenous TC45, 14-3-3σ, β-actin, or Lamin A/C (nuclear protein loading control).
